# Preface

**Published:** 2009-10

**Authors:** Jyotsna Murthy

**Affiliations:** Guest Editor, Cleft Supplementary, Indian Journal of Plastic Surgery

Cleft lip and palate is not a only medical and health problem, but a social problem as well. Hence it needs to be assesssed, understood and practiced keeping all these aspects into consideration. Though its social implications are yet to be studied scientifically in the developing world, we do the best in our capacity to improve the quality of services and reduce the morbidity.

Little has been understood about the facial growth and therefore cleft lip and palate remains a mystery. Management of cleft lip and palate has more controversies than consensus! Children with cleft lip and palate can be rehabilitated easily in society by proper treatment. However, the morbidities associated with it are well known starting from surgical morbidity like fistula and poor nasolabial appearance, inadequate speech and dental rehabilitation and psychosocial morbidities that go hand in hand throughout childhood and adolescence. This special issue of IJPS will throw light on these subjects so that readers are able to gain some technical point t to improve, treatment and protocol.

New advances have three phase of introduction-enthusiastic application, studying outcome and result and building up of consensus for correct indications. Preoperative moulding of arches is known for ages, but presently it is expanded to active manipulation with moulding the nasal cartilages - Naso-Alveolar Moulding (NAM). Though the procedure is attractive, but it is not above controversy and the long term effect is not very encouraging. The introduction of distraction osteogenesis for facial skeleton by Dr. J. McCarthy has provided a useful procedure to tackle severely retruded faces which was difficult to manage by routine orthognathic surgery earlier. Midface distraction is of great help in younger patients and patients with severely retruded face to improve occlusion, function and facial harmony.

We hope to find the solution to cleft anomaly through genetics! Easier said than done, The combined strength of scientists researching cleft genetics shall surely throw light on aetiology, effects and facial growth; and will guide us in planning cleft treatments in future.

In spite of all our combined efforts, good intentions and advances, some patients still receive suboptimal care resulting in increasing morbidity in these children. It is our duty to provide complete rehabilitation and relentlessly strive towards affording the best care for the cleft patient. This supplementary issue is one of such efforts towards improving our understanding of holistic cleft care.

I hope you like reading it.

